# Antibiotic resistance and pathogenicity assessment of various *Gardnerella* sp. strains in local China

**DOI:** 10.3389/fmicb.2022.1009798

**Published:** 2022-09-26

**Authors:** Kundi Zhang, Mengyao Lu, Xiaoxuan Zhu, Kun Wang, Xuemei Jie, Tan Li, Hongjie Dong, Rongguo Li, Fengyu Zhang, Lichuan Gu

**Affiliations:** ^1^State Key Laboratory of Microbial Technology, Shandong University, Qingdao, China; ^2^Jinan Key Laboratory of Female Reproductive Tract Infection, Jinan Genital Tract Microecological Clinical Laboratory, Jinan Maternity and Child Care Hospital Affiliated to Shandong First Medical University, Jinan, China; ^3^Faculty of Health Sciences, Cumming School of Medicine, Calgary, AB, Canada

**Keywords:** *Gardnerella vaginalis*, prevalent strains, comparative genomics, antibiotic resistance, accurate diagnosis and therapy

## Abstract

*Gardnerella* overgrowth is the primary cause of bacterial vaginosis (BV), a common vaginal infection with incidences as high as 23–29% worldwide. Here, we studied the pathogenicity, drug resistance, and prevalence of varying *Gardnerella* spp. We isolated 20 *Gardnerella* strains from vaginal samples of 31 women in local China. Ten strains were then selected *via* phylogenetic analysis of *cpn60* and *vly* gene sequences to carry out genome sequencing and comparative genomic analysis. Biofilm-formation, sialidase, and antibiotic resistance activities of the strains were characterized. All strains showed striking heterogeneity in genomic structure, biofilm formation and drug resistance. Two of the ten strains, JNFY3 and JNFY15, were classified as *Gardnerella swidsinskii* and *Gardnerella piotii*, respectively, according to their phenotypic characteristics and genome sequences. In particular, seven out of the ten strains exhibited super resistance (≥ 128 μg/mL) to metronidazole, which is the first line of treatment for BV in China. Based on the biochemical and genomic results of the strains, we proposed a treatment protocol of prevalent *Gardnerella* strains in local China, which provides the basis for accurate diagnosis and therapy.

## Introduction

*Gardnerella vaginalis* is a facultatively anaerobic bacterium of the *Bifidobacteriaceae* family and part of the normal vaginal microbiome. Often described as a Gram variable organism with a Gram-positive wall type, the genome size of the type strain ATCC 14018 is remarkably small compared to other facultative anaerobes (Greenwood and Pickett, 1980). At only 1.6 M, it is one-third and one-fifth that of *E. coli* and *Pseudomonas aeruginosa*, respectively. In addition, no plasmids nor phages have been characterized in any identified strains thus far (Yeoman et al., 2010). As such, the metabolic pathway of *G. vaginalis* is also relatively simple, with only succinic acid dehydrogenase and malate dehydrogenase remaining in the TCA cycle (Greenwood and Pickett, 1980).

As a conditional pathogen, *G. vaginalis* has a very simple cell signaling system and a relatively rich virulence system, which leads to differences in pathogenicity amongst various strains. The type strain, for example, does not contain signaling molecules such as c-di-NMP and cNMP (He et al., 2016), thus lacking the second messenger system of cyclic nucleotides commonly used in most strains. It only has six pairs of two-component systems (TCS), which is rather simple compared to the 130 pairs of TCS in *Pseudomonas aeruginosa* (Francis et al., 2017). Regarding the virulence system, strain ATCC 14019 has no typical secretory system (Hood et al., 2010) and does not contain flagella, and its adhesion to the epithelial cell mainly relies on type I and type II pili (Punsalang and Sawyer, 1973). It secretes vaginolysin, a cholesterol-dependent cytotoxin (Rottini et al., 1990; Gelber et al., 2008). As for other genes associated with pathogenicity, it has two high-affinity iron transporters and is resistant to bleomycin, methicillin and lanthionine antibiotics (Berger-Bachi et al., 1989; Jarosik et al., 1998). The mechanism of immune escape mainly involves changing the molecular weight of its surface antigen (Lindahl et al., 2005). Previously, it has been observed that *G. vaginalis* can form a dense biofilm structure and stick to the surface of the vaginal epithelial cells, resulting in stubborn drug resistance (McGregor and French, 2000; Workowski et al., 2010; Rosca et al., 2020).

Although *G. vaginalis* commonly occurs in the vaginal microbiota of healthy individuals (Fredricks et al., 2005; Hyman et al., 2005; Kim et al., 2009), it is also one of the most frequent and predominant vaginal tract colonists in women diagnosed with bacterial vaginosis (BV) (Fredricks et al., 2005; Menard et al., 2010; Patterson et al., 2010). BV is the most common bacterial inflammatory reproductive tract disease amongst females of reproductive age, with incidence rates as high as 23–29% worldwide (Peebles et al., 2019). BV results in a range of symptoms such as vaginal itching, odor, and abnormal vaginal discharge, and it is caused by the disruption of the dynamic balance between the bacterial microflora in the vagina, the host, and the environment. This involves the overgrowth of various anaerobic bacteria in the vaginal microbiome relative to *Lactobacilli*, with *G. vaginalis* overgrowth in particular being implicated in the formation of a stubborn biofilm that is indicative of BV (Anahtar et al., 2018; Rosca et al., 2020). This biofilm can adhere to the vaginal epithelium, forming clue cells for the diagnostic basis of BV (Swidsinski et al., 2005). Additionally, *G. vaginalis* infections have been associated with various clinical presentations. Many *G. vaginalis* infections can cause pelvic inflammatory disease, salpingitis, infertility, and gynecological tumors. *G. vaginalis* infections have also been associated with adverse outcomes in pregnancy—incidences of premature rupture of membranes, premature delivery and intrauterine infection were 25, 31.67, and 32%, respectively, which were significantly higher than those in normal pregnant women (Cauci et al., 2008; Gelber et al., 2008; Fettweis et al., 2019).

In recent years, comparative genomics studies on *G. vaginalis* have emerged. Whole genome DNA-DNA hybridization of vaginal samples from women presenting varying phenotypes led to a reclassification of several *G. vaginalis* strains (UGent 06.41, UGent 18.01, GS9838-1). These strains have been reclassified to *G. leopoldii, G. piotii*, and *G. swidsinskii*, respectively (Vaneechoutte et al., 2019). Further genome sequencing and comparative analyses of three *G. vaginalis* strains (ATCC 14018, ATCC 14019, and 409-05) showed the high genetic diversity of this species, with only 846 genes out of more than 1,300 genes in the genome being identical. All three strains are able to thrive in vaginal environments, thus allowing the BV isolates ATCC 14018 and 14019 to occupy a niche that is unique from 409-05. Each strain has significant virulence potential, although genomic and metabolic differences, such as the ability to degrade mucin, indicate that the detection of *G. vaginalis* in the vaginal tract provides only partial information on the physiological potential of the organism (Yeoman et al., 2010). Comparative genomic analyses of strains 5-1 (from healthy hosts) and AMD (from bacterial vaginosis) showed that the copy number and amino acid sequence of vaginolysin in these two strains were almost identical, with only one amino acid difference. Strain AMD contains more toxin-antitoxin (TA) systems; Strain 5-1 lacks two key adhesion proteins, Rib, significantly reduced ability to adhere to epithelial cells, and is more sensitive to erythromycin, leading to weakened pathogenicity (Harwich et al., 2010). All these studies have provided a solid basis for the development of novel diagnostics and treatments against *Gardnerella* infections. However, given the high heterogenicity of *Gardnerella*, and the large population with high BV infection rates in China, the lack of in-depth research on varying *Gardnerella* strains poses a concern for the treatment of patients in China.

Therefore, this study investigates ten epidemic strains of *Gardnerella* in local China. First, we used molecular biology and biochemistry techniques to classify and determine the epidemic percentage of each subtype. Then, we analyzed both common and unique pathogenic genes of different *Gardnerella* strains through comparative genomics. Based on these results, we constructed a database of epidemic *Gardnerella* strains in local China, which may act as a foundation for developing accurate diagnostics and therapeutics for *Gardnerella-*induced BV.

## Materials and methods

### Selection of patients

Samples were obtained from 31 women who attended private gynecology clinics in Jinan Maternal and Child Health Care Hospital, China. The study was approved by the Jinan Health Committee (approval no. 2019-1-25). Written informed consent was obtained from all study participants prior to enrollment. All were Han Chinese > 18 years of age (range, 21–55 years; mean, 31.2 years). All had come to the clinic for a routine gynecological examination, with self-reported complaints of vaginal itching/burning sensations, or with increased and/or malodorous vaginal discharge. All participants were asked to complete a questionnaire on the current use of hormonal contraceptives, menstrual cycle, and frequency of vaginal infections. Exclusion criteria included menstruation at the time of enrollment, human immunodeficiency virus (HIV) infection, and antibiotic/antimicrobial treatment within 14 days of sampling.

### Examination of vaginal samples, *Gardnerella vaginalis* isolation

All samples were subjected to Gram-staining and microscopy to assess their Nugent score (NS) (Nugent et al., 1991). BV diagnosis was also defined by the clinician. BV diagnosis included the mandatory satisfaction of three out of the four Amsel criteria (elevated pH, clue cells, fishy odor, and characteristic vaginal discharge); this was supplemented by chemical analysis results (H_2_O_2_ concentration, activity of leucocyte esterase, sialidase, coagulase and beta-glucosidase) (Amsel et al., 1983; Workowski et al., 2010). A sample was considered as BV-positive if the NS ranged from 7 to 10 and at least three Amsel criteria were present (Workowski et al., 2010). In the case of any inconsistency between the results of chemical analysis and morphology, it should be subjected to morphology. For *G. vaginalis* isolation, a swab taken near mid-vagina was placed in a BHI liquid medium and then the 10 × gradient dilution was spread on a Chocolate Agar Medium (Haibo, Qingdao, China). Chocolate agar plates were incubated at 37^°^C in 5% CO_2_ for 48 h. Colonies of *G. vaginalis* were identified as described previously (Pleckaityte et al., 2012).

### Growth condition

Planktonic cells were grown in sBHI [Brain-heart infusion supplemented with 2% (wt/wt) gelatin (Aladdin, Shanghai, China), 0.5% (wt/wt) yeast extract (Oxford, UK), 0.1% (wt/wt) glucose and 0.1% (wt/wt) soluble starch (Aladdin, Shanghai, China)] for 24 h at 37°C with 5% CO_2_. For biofilm formation, the glucose was replaced by maltose, and 5% (v/v) goat blood was added. 2% mid-log phase seed was inoculated to a fresh medium for the following tests.

### Gene-specific PCR assays and phylogeny construction

*Gardnerella vaginalis* identification was confirmed by PCR amplification of the 16S rRNA gene (Rainey et al., 1996) and sequencing of the obtained PCR product. *G. vaginalis* subtypes were classified through phylogenetic tree construction using *cpn60* and *vly* gene sequences according to previous studies (Janulaitiene et al., 2017, 2018). The primers are listed in Supplementary Table 1.

### Sialidase assay

To further characterize the virulence factors of *Gardnerella* clinical isolates, we investigated the presence and expression of the sialidase gene. The presence of the sialidase gene in clinical isolates of *Gardnerella* was identified by PCR using specific primers (Supplementary Table 1). In addition, The *Gardnerella* cultures were diluted to an OD_600_ value of 0.8 with 50 mM MES buffer (pH 5.5). The reaction system contained 2.2 mM NBT (Nitrotetrazolium Blue chloride, Sigma-Aldrich, St. Louis, MO), 146 mM sucrose, 10.5 mM MgCl_2_, 6.3 mM BCIN (5-Bromo-4-chloro-3-indolyl α-D-N-acetylneuraminic acid sodium salt, Sigma-Aldrich, St. Louis, MO), with 0.5% sulfonyl 440 added to avoid bubbles. The mixture was then incubated at 37°C for 20 min and 100 μL of the incubated mixture was added to the wells of black polystyrene microplates (Nunc, Thermo Fisher Scientific). The plates were sealed with an optically clear seal, and BCIN hydrolysis was monitored by measuring the fluorescence at a wavelength of 616 nm using a SynergyH4 hybrid multi-mode microplate reader (Biotek, Winooski, VT, USA) (Zhang and Rochefort, 2013; Janulaitiene et al., 2018). The fluorescence of each supernatant was analyzed in triplicate.

### Biofilm assay

For biofilm formation, the cell concentration of 24 h-old cultures was assessed by measuring the optical density of the cultures at 600 nm (Model Sunrise, Tecan, Switzerland). The cultures were further diluted to obtain a final concentration of approximately 10^6^ CFU/mL. After homogenization, 200 μL of *G. vaginalis* suspensions were dispensed into each well of three 96-well flat-bottom tissue culture plates (Orange Scientific, Braine L’Alleud, Belgium). The tissue culture plates were incubated at 37°C in 10% CO_2_. After 24 h, the culture medium covering the biofilm was removed, then replaced by fresh sBHI and allowed to grow under the same conditions for an additional 24 h. The biofilm produced was quantified by crystal violet staining and then scanned at OD 570 (Jayaprakash et al., 2012; Janulaitiene et al., 2018).

### Antibiotic resistance

All antibiotic reserve fluids were prepared at a concentration of 5,120 μg/mL, filtered by a filter membrane to ensure sterility, and stored separately at –20°C. The final antimicrobial concentration was obtained by double dilution with sBHI broth, which was diluted to 512, 256, 128, 64, 32, 16, 8, 4, 2, 1, 0.5, 0.25, and 0.125 μg/mL, respectively. The microdilution plate was made of a 96-well plate with 100 μL of prepared working fluid added to each well. At least one well containing only 100 μL of the antimicrobial-free broth was used as a growth control for the test strain. At the same time, at least one well containing only 100 μL of antimicrobial-free broth was used as an unvaccinated negative control well. Three to five colonies were selected from the blood plate medium with a sterile inoculation loop and placed in a sBHI liquid medium. The broth was incubated at 37^°^C, until it reached a turbidity of at least 0.5 McFarland (spectrophotometer 625 nm wavelength, 1 cm path, absorption rate 0.08–0.13). To maintain the stability of the cell concentration in the inoculation suspension, the microdilution plate must be inoculated within 30 min after preparation of the inoculation suspension. In the microdilution plate containing 100 μL of diluted antimicrobial agents, 5 μL of cell suspension was added to each well so that the number of cells in each well was approximately 5 × 10^5^ CFU/mL. The microdilution plate was placed in an incubator at 37^°^C and 5% CO_2_ for 18 ± 2 h. When the bacteria in the growth control hole had sufficient growth and the negative control hole without inoculation did not grow, MIC was determined as the minimum drug concentration that could significantly inhibit bacterial growth (Clinical and Laboratory Standards Institute, 2020a,b).

### Genome sequencing

The strains were cultured to the middle and late logarithmic stage, and cells were collected. The QIAGEN Genomic DNA extraction kit (QIAGEN, Dusseldorf, Germany) was used for Genomic DNA extraction of the samples according to the standard operating procedure provided by the manufacturer. The extracted genomic DNA was determined with the NanoDrop One UV-vis spectrophotometer (Thermo Fisher Scientific, Massachusetts, USA). OD260/280 was within 1.8–2.0, and OD 260/230 was between 2.0 and 2.2. DNA was subsequently quantified using the Qubit 3.0 Fluorometer (Invitrogen, California, USA).

After quality inspection, the Blue Pippin automatic nucleic acid recycling instrument (Sage Science, Massachusetts, USA) was used to cut and recycle large fragments. Then, the connectors in the LSK108 linking kit (Oxford, UK) were used for the linkage reaction. Finally, the Qubit 3.0 Fluorometer (Invitrogen, California, USA) was used for accurate quantitative testing of the established DNA libraries. After completion of database construction, a DNA library of a certain concentration and volume was added into one flow cell, and the flow cell was transferred to the Nanopore GridION X5 (Oxford Nanopore Technologies, UK) for real-time single-molecule sequencing.

### Genome analysis and phylogenomic tree construction of *Gardnerella* taxon

After quality control, the data was assembled with CANU^1^ and corrected with PILON (Walker et al., 2014) combined with second-generation sequencing data. The corrected genome uses its own script to detect whether it is ringed or not. After the redundant parts are removed, the origin of the ringed sequence is moved to the replication starting site of the genome with Circlator (parameter: Fixstart) to obtain the final genome sequence.

The coding gene was predicted by Prodigal^2^ and the complete CDS was retained. The tRNA gene was predicted by Transcan-SE, and the rRNA gene was predicted by RNAmmer. Other ncRNA searched the RFAM database for predictions using Infernal, retaining the predicted length (Kalvari et al., 2020). CRISPR was predicted with MinCED (Bland et al., 2007) and Islander was predicted with IslandViewer.^3^

Genome-encoded proteins were extracted and annotated with InterPro^4^ to extract annotation information from TIGRFAMs, Pfam and GO databases. Blastp was used to compare the coded proteins to KEGG and Refseq databases, and the best result of the comparison coverage > 30% was kept as the annotation result. The encoding protein was compared with the COG database for COG annotation by RPSBLAST. ABRicate could obtain resistance genes from contig, which correlated with databases such as NCBI, CARD [The Comprehensive Antibiotic Resistance Database (mcmaster.ca)] and ARG-Annot (Gupta et al., 2014). ABRicate software was also used to predict the resistance genes in the genome. Sequencing strain genes were compared with the Pathogenic Bacteria Virulence factor database (VFDB).^5^ The VFDB database, developed by the Chinese Academy of Medical Sciences, is widely used in the identification of virulence factor genes. VFDB collected the sequence information of bacterial virulence genes from 30 genera (74 pathogens). The bacterial genes were predicted using AntiSMASH.^6^ AntiSMASH used a rules-based clustering method to identify 45 different types of secondary metabolite biosynthesis pathways through its core biosynthesis enzyme.

To assess genome differences between *Gardnerella* strains, a phylogenetic analysis involving 22 genome sequences retrieved from NCBI and our data was performed. For this purpose, the genome sequences were aligned using MAFFT, and the phylogenetic tree was constructed using the neighbor-joining method in Clustal W v2.1; the image was produced using FigTree software.^7^

## Results

### Collection and phylogenetic analysis of *Gardnerella* isolates

**FIGURE 1:**
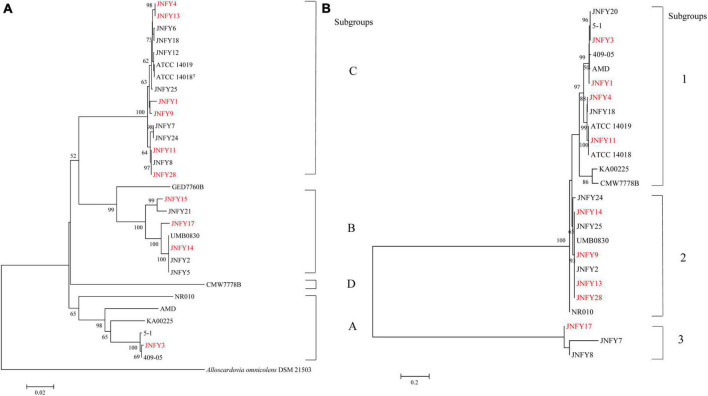
Phylogenetic analysis of *Gardnerella* strains based on *cpn60*(a) and *vly*(b) sequences. **(A)** Phylogenetic tree of *Gardnerella cpn60* sequences comprising four distinct clades: (A–D). Bootstrap values for each node are indicated. ATCC 14018^T^, ATCC 14019, 41V, 101, 1500E, AMD, 409-05, JCP8066, and JCP7719 are *G. vaginalis* isolates with whole genome sequence information available in Genbank (Accession numbers ADNB00000000, CP002104, AEJE00000000, AEJD00000000, GCA_000263595, ADAM00000000, CP001849, GCA_000414565, and GCA_000414625, respectively). **(B)** Phylogenetic tree of *Gardnerella vly* sequences comprising four distinct clades: 1, 2, 3, and 4 (no *vly* gene).

Twenty *Gardnerella* clinical strains were isolated from characterized vaginal samples of 31 women in local China. Each plate was inoculated from a single vaginal swab. *Gardnerella* strains were identified as described in Methods. Isolates from individual colonies were then subtyped by clade-specific PCR. Based on *cpn60* gene sequences, 13 isolates were defined as belonging to clade C, six isolates belonged to clade B, and one isolate belonged to clade A. We found no *Gardnerella* strains belonging to clade D (Figure 1A). Two isolates (JNFY3 and JNFY13) originated from vaginal samples with normal vaginal microflora (NS = 1 and 0, respectively), whereas the other 29 vaginal swabs with NS values ranging from 4-10 (Table 1). The 20 *Gardnerella* clinical strains were divided into four clades based on their *vly* gene sequences (Figure 1B), and strains without the *vly* gene (JNFY15 and JNFY21) were classified as subtype 4.

**TABLE 1:** Polyphasic comparison of the *Gardnerella* strains.

Strains	Nugent score	Biofilm intensity	Phylogenetic subgroups	to Mid-log phase (h)	Sialidase activity	Vaginolysin-encoding gene	Antibiotic resistance genes	MID = 16 μ g/ml
ATCC 14019	ND	1.69	C1	10 h	ND	+	*lsaC*	GM, TO
JNFY1	4	1.42	C1	14 h	0.29±0.009	+	*ermX*, *lsaC*	CIP, TO, MTZ
JNFY3	1	0.48	A1	8 h	ND	+	*ermX*	AZM, CIP, GM, TO, MTZ
JNFY4	8	1.06	C1	9 h	ND	+	*ermX*, *lsaC*, *tetL*, *tetM*	AZM, TE, CIP, GM, ERM, TO, CLI, MTZ
JNFY9	7	1.03	C2	15 h	ND	+	*ermX*, *lsaC*, *tetL*, *tetM*	TE, CIP, TO
JNFY11	8	1.29	C1	10 h	ND	+	*ermX*, *lsaC*	CLI, CIP, TO, AZM
JNFY13	0	1.1	C2	12 h	ND	+	*lsaC*, *tetL*, *tetM*	GM, TE, TO, MTZ
JNFY14	8	2.57	B2	11 h	0.38±0.008	+	2*lsaC	CIP, GM, TO, MTZ
JNFY15	8	0.59	B4	20 h	ND	-	*ermX*, *lsaC*	CIP, GM, ERM, CLI, TO
JNFY17	4	0.52	B3	13 h	0.27±0.014	+	*lsaC*	CIP, GM, TO, MTZ
JNFY28	8	1.13	C2	14 h	ND	+	*lsaC*	GM, DNR, TO, MTZ

### Biofilm, biomass, and sialidase activity in *Gardnerella* strains

**FIGURE 2:**
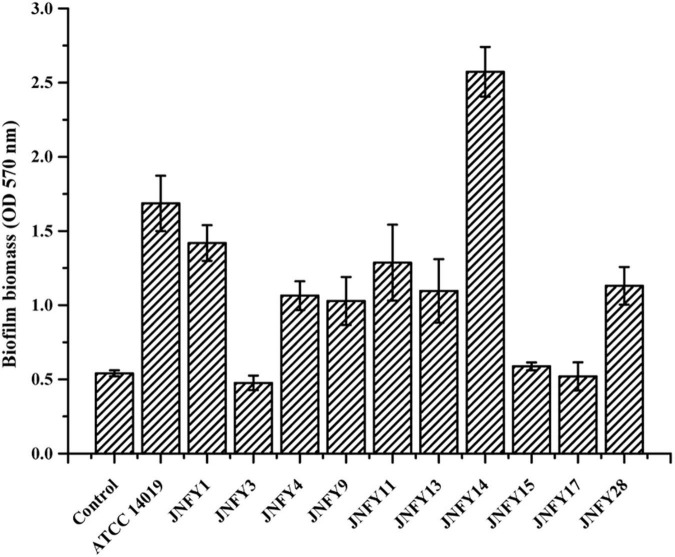
Biofilm formation by *Gardnerella* strains. Isolates were cultured in 96-well plate in BHI medium, stained at 48 h, after removal of planktonic cells.

The measurement of biofilm formation capacity of 10 strains showed that, except for JNFY3, JNFY15, and JNFY17, other prevalent strains could form biofilm with varied thickness. JNFY14 produced the largest amount of biofilm, and *cpn60* subtype C prevalent strains generally formed a certain amount of biofilm (Table 1, Figure 2, and Supplementary Figure 2). Sialidase activity was detected in strains JNFY3, JNFY4, JNFY11, and JNFY17. Moreover, strain JNFY14 exhibited weak sialidase activity (Table 1).

### General genome features and phylogenomic tree of *Gardnerella* strains

The genome size of *Gardnerella* was between 1.5 and 1.8 Mb, which was smaller than other members of the *Bifidobacteriaceae* family, one-third the size of *E. coli* and one-fifth the size of *P. aeruginosa*. The genomes of these ten strains contained about 1,300 genes, with a GC content of about 41–43%. The basic features of the ten prevalent GV strains were similar to those of the American strain ATCC 14019 isolated from BV patients (genome size 1,667,350 bp, GC content 41%). No obvious plasmid sequence was found in any of the ten strains. One genomic island was predicted in JNFY1, JNFY11, and JNFY17, and two genomic islands were predicted in JNFY3. No genomic island was predicted in other strains. A CRISPR sequence was predicted in JNFY1, JNFY3, JNFY4, JNFY13, and JNFY28, but not in other prevalent strains (Table 2). The phylogenomic tree showed that the *Gardnerella* strains were generally divided into four groups, which was similar to the *cpn60* phylogenetic tree (Figure 1A). Strains JNFY1, 4, 9, 11, 13, 28, and ATCC 14019 were located in the same group in both trees; likewise, strains 409-05, JNFY3, 5-1, and JNFY15, 17, UMB0830 located to the same groups. Strains JNFY3 and JNFY15 were classified as *Gardnerella swidsinskii* and *Gardnerella piotii*, respectively, according to their genome sequences (Supplementary Figure 1). On the basis of the distance from the position of each strain to the root of the phylogenomic tree (Supplementary Figure 1), we calculated that the evolutionary order of each strain is as follows: ATCC 14018, JNFY3, 409-05, 5-1, JNFY14, JNFY15, JNFY17, JNFY4, JNFY11, JNFY9, JNFY13, JNFY1, and ATCC 14019.

**TABLE 2:** The general features of the 10 *Gardnerella* genomes.

Strains	Size (bp)	CDS number	Plasmid	Island	GC %	tRNA	rRNA	CRISPR	Accession No.
JNFY1	1,711,437	1,316	NO	1	41.61	45	6	1	CP083177
JNFY3	1,602,355	1,245	NO	2	42.02	45	6	1	CP083176
JNFY4	1,742,450	1,359	NO	NO	41.56	45	6	1	CP083175
JNFY9	1,640,523	1,243	NO	NO	41.41	45	6	NO	CP083174
JNFY11	1,743,456	1,357	NO	1	41.82	45	6	NO	CP083173
JNFY13	1,682,566	1,290	NO	NO	41.64	45	6	1	CP083172
JNFY14	1,680,963	1,290	NO	NO	41.67	45	6	NO	CP083171
JNFY15	1,541,442	1,164	NO	NO	42.55	45	6	NO	CP083170
JNFY17	1,595,814	1,236	NO	1	42.74	45	6	NO	CP083169
JNFY28	1,542,082	1,392	NO	NO	42.23	45	6	1	CP083168

### Carbohydrate transport and metabolism

In general, biofilm formation is tightly related to carbohydrate transport and metabolism. Compared with the biofilm-rich strain JNFY14, the biofilm-lacking strains JNFY3, JNFY15, JNFY17, and JNFY28 lacked many genes involved in polysaccharides synthesis and sugar transport. Absent genes included *afuC*, *araD*, *fucP*, *galM*, *galT*, *lacZ*, *mglA*, *xylB*, and *xylF*. In addition, there were fewer copies of *nagC*, *ugpA*, *ugpB*, and *ugpE* in biofilm-lacking strains, compared to the biofilm-rich strain JNFY14 (Supplementary Table 2). The absence and reduced copy numbers of these genes may explain why the strains of this group cannot form normal biofilm.

### Antibiotic resistance

According to the genomic data, all of the ten *Gardnerella* isolates contained antibiotic resistance genes, with a total of four detected antibiotic resistance genes. JNFY1, JNFY3, JNFY4, JNFY9, JNFY11, and JNFY15 contained the macrolide erythromycin resistance gene *ermX*. Strains JNFY1, JNFY4, JNFY9, JNFY11, JNFY13, JNFY14, JNFY15, JNFY17, and JNFY28 contained the lincosamide antibiotic resistance gene *lsaC*, while strains JNFY4, JNFY9, and JNFY13 contained the tetracycline resistance genes *tetL* and *tetM*. The comparative genomics data also showed that all strains contain the daunorubicin resistance protein (Table 3).

**TABLE 3:** Antibiotic-resistance genes predicted in genome of the *Gardnerella* strains.

Strains	Macrolides-resistant		Tetracyclines-resistant	
	*ermX*	*lsaC*	*tetL*	*tetM*
ATCC 14019	0	1	0	0
JNFY1	1	1	0	0
JNFY3	1	0	0	0
JNFY4	1	1	1	1
JNFY9	1	1	1	1
JNFY11	1	1	0	0
JNFY13	0	1	1	1
JNFY14	0	2	0	0
JNFY15	1	1	0	0
JNFY17	0	1	0	0
JNFY28	0	1	0	0

Resistance tests showed that *Gardnerella* strains containing erythromycin resistance genes were resistant to macrolide azithromycin. Similarly, prevalent strains containing tetracycline resistance genes were resistant to tetracycline. Strains JNFY13 and JNFY28 showed weak resistance to erythromycin, despite their lack of *ermX*. Alternatively, strain JNFY3 showed strong resistance to lincosamide, despite its lack of *lsaC*. YadH (ABC-type multidrug transport system) and McrA (5-methylcytosine-specific restriction endonuclease) existed only in metronidazole-resistant strains JNFY3, JNFY4, JNFY14, JNFY17, and JNFY28 (Table 4), thus these genes are likely involved in metronidazole resistance.

**TABLE 4:** MIC (minimum inhibitory concentration) of the *Gardnerella* strains on common antibiotics in clinic.

Antibiotics-resistant genes	MIC(μg/mL)											
	First-line								Second-line			Third-line
	Beta-lactam	Tetracycline	Macrolide			Quinolone	Nitroimidazole	Aminoglycoside	Macrolide	Aminoglycoside	Beta-lactam	Glycopeptide
	AMP	TE	AZM	ERM	CLI	CIP	MTZ	GM	DNR	TO	CMR	VA
ATCC 14019	<0.25	<0.25	<0.25	0	0	2	8	16	2	64	0.25	1
JNFY1	0.5	0.25	4	0.5	0	16	128	8	0	16	0.25	1
JNFY3	0.5	0.25	16	0.125	0.0625	16	>128	16	8	32	0.5	1
JNFY4	0.5	16	16	32	128	16	>128	16	2	32	0.5	1
JNFY9	0.25	16	8	0	0	16	4	4	0.125	16	0.5	1
JNFY11	1	0.25	64	0.125	16	16	2	8	1	32	1	1
JNFY13	0.25	32	0.25	0.125	0	1	>128	16	2	128	1	1
JNFY14	4	0⋅125	0.5	0	0.0625	32	>128	32	0⋅5	32	1	1
JNFY15	0.5	<0.25	8	128	128	16	16	32	1	128	0.5	0.5
JNFY17	4	0.5	0.5	0	0	32	>128	32	0	64	2	1
JNFY28	0.5	0.25	0.25	0.25	0	8	>128	16	32	128	0.5	1

AMP, ampicillin; TE, tetracycline; AZM, azithromycin; ERM, erythromycin; CLI, clindamycin; CIP, ciprofloxacin; MTZ, metronidazole; GM, gentamicin; DNR, daunorubicin; TO, tobramycin; CMR, chloramphenicol; VA, vancomycin.

### Virulence

Typically, epithelial cell adhesion is mediated by pili. All ten strains of *Gardnerella* contained *ppK*, a gene encoding for type IV pili. Some *Gardnerella* strains share virulence factors involved in pathogenic mechanisms like epithelial cell adhesion, iron absorption, secretion, acting as toxins and endotoxins, and immune escape; virulence factors can also have regulatory functions (Supplementary Table 3). Strains lacking these virulence factors such as JNFY3, JNFY15, and JNFY17 have the common feature of impaired biofilm formation. *Gardnerella* produces vaginolysin, which plays a critical role in BV pathogenesis. The gene encoding vaginolysin was detected in nine strains, with the exception of JNFY15 (Supplementary Table 4). Strains JNFY3 and JNFY4 exhibited nine and one unique virulence factor(s), respectively, with virulence factors having roles in allantoin utilization, adhesion, secretion and iron uptake (Supplementary Table 5). The TA components and other competitive exclusion genes of the *Gardnerella* strains are listed in Table 5. There are mainly four TA systems in *Gardnerella* genomes including HicAB family, PHD-RelE family, ParE family, and RelB-Txe family, with various distribution in the 11 strains. However, the other genes related to competitive exclusion showed a similar pattern in each strain.

**TABLE 5:** TA system components and other competitive exclusion genes.

TA system	ATCC 14019	JNFY1	JNFY3	JNFY4	JNFY9	JNFY11	JNFY13	JNFY14	JNFY15	JNFY17	JNFY28
HicA-family TA system toxin HicB-family TA system antitoxin	1	2	HicB only	n/a	1	2	n/a	2	1	1	n/a
PHD/YefM family TA system antitoxin RelE/StbE family TA system toxin	1	n/a	PHD only	PHD only	n/a	PHD only	n/a	PHD only	1	PHD only	n/a
ParE-family TA system toxin TA system antitoxin	3	1	1	n/a	1	n/a	1	ParE only	1	2	n/a
RelB/DinJ family TA system antitoxin Txe/YoeB family TA system toxin	5	1	3	RelB only	RelB only	1	RelB only	1	n/a	RelB only	1
**Other genes with potential roles in competitive exclusion**											
Abi-like protein	n/a	3	1	n/a	n/a	n/a	n/a	n/a	2	2	2
CHAP domain protein	2	2	2	2	2	2	2	2	2	2	3
GH25 enzyme	1	1	1	1	1	1	1	1	1	1	1
SalY-family antimicrobial peptide ABC transport system, ATP-binding protein	4	5	5	5	5	5	5	5	6	5	5

n/a indicates protein was not identified within the genome.

### Secondary metabolites biosynthesis

Analysis of secondary metabolite gene clusters in ten isolates of *Gardnerella* demonstrated the presence of polyketosynthase (PKS)-related secondary metabolite gene clusters in the *Gardnerella* genome. PKS genes encode an enzyme, or enzyme complex with multiple domains, capable of synthesizing polyketo compounds, including common antibiotics such as erythromycin, tetracycline and so on. Type I, type II, and type III PKS synthesize T1PKS, T2PKS, and T3PKS, respectively. The T3PKS gene cluster was predicted in all strains except JNFY3.

## Discussion

Bacterial vaginosis (BV) is a common vaginal infection in women of child-bearing age, with *Gardnerella* spp. being the main pathogen of BV. Alongside BV, *Gardnerella* spp. has also been associated with vertebral osteomyelitis and discitis (Graham et al., 2009), retinal vasculitis (Neri et al., 2009), acute hip arthritis (Sivadon-Tardy et al., 2009), and bacteremia (Chen et al., 2018). While the vaginal overgrowth of several anaerobic bacteria has been associated with BV, *Gardnerella* spp. has a stronger adhesion to vaginal epithelium and a stronger tendency to form biofilm compared to other BV-related microorganisms. It can be used as a scaffold to attach other anaerobic bacteria and is associated with the onset and recurrence of BV (Anahtar et al., 2018). The treatment of recurrent BV is difficult, and existing treatment measures include the prolongation of an antibiotic course and consolidation treatment. However, due to biofilm formation and strain differences in *Gardnerella*, antibiotics cannot eliminate the bacteria, making the current treatment of BV unable to achieve ideal therapeutic effect (Cohen et al., 2020; Laniewski et al., 2020). Therefore, it is of great significance to construct a database detailing prevalent *Gardnerella* strains in local China, which can provide the basis for the accurate diagnosis and therapy of BV and allow for the development of more effective antibacterial drugs targeting prevalent strains.

In this study, 20 prevalent strains of *Gardnerella* from local China were collected from clinical specimens to study the differences in their pathogenicity and drug resistance. This would provide a theoretical basis for the accurate diagnosis and treatment of BV. First, based on *cpn60* sequence genotyping, the 20 prevalent strains were divided into subtypes A, B, and C. Based on *vly* sequence genotyping, prevalent strains were divided into subtypes 1, 2, 3, and 4. Ten strains of subtypes C1 (JNFY9, 13 and 28), C2 (JNFY1,4 and 11), B1 (JNFY14 and 17), A1 (JNFY3), and B4 (JNFY15) were selected for comparative genomics. The genome size of the 10 strains ranged from 1.54 to 1.74 Mb, and the GC content was about 41–43%. No plasmid was observed, and 1–2 gene islands were predicted in 4 strains. The phylogenomic tree of the *Gardnerella* strains showed that strains JNFY3, JNFY15,17, and JNFY1,4,9,11,13,14,28 were located in three different branches. This was almost identical to the *cpn60* phylogenetic tree, except for the position of strain JNFY14. Therefore, the *cpn60* distribution could represent genomic classification to some extent. The *G. vaginalis* strains 5-1, 409-05 and JNFY3 were grouped in the same branch in all three trees, further indicating the weak pathogenicity of these strains in healthy hosts.

Virulence factors associated with microbial pathogenesis were found in the genome of prevalent strains, including factors for adhesion, secretion, iron and magnesium absorption, immune escape, and toxins. Common virulence factors were found in the ten prevalent strains, although differences also exist amongst the strains. It is worth noting that JNFY3 had nine specific virulence factors while JNFY4 had one. In addition, JNFY3, JNFY15, and JNFY17 had severely hindered abilities in biofilm formation. These three strains not only lacked certain carbohydrate metabolism genes (Supplementary Table 2), but also lacked virulence factors related to adhesion, iron absorption and toxins, which might be conductive to biofilm formation in the comparative genomic analysis.

Subsequent biochemical experiments were conducted to preliminarily investigate the pathogenic mechanism of *Gardnerella*. Experiments on the biofilm-forming capabilities of *Gardnerella* showed variable abilities to form biofilm amongst different prevalent strains with *cpn60* subtype C displaying weak to moderate biofilm-forming abilities. Sialidases are enzymes associated with bacterial invasion of the host and are implicated as virulence factors in diseases such as meningitis, glomerulonephritis, and periodontal disease (Corfield, 1992). Previous studies have shown that BV-associated bacteria produce sialidase, and its activity is inversely related to vaginal IgA response against vaginolysin produced by *Gardnerella* (Cauci et al., 2003). The sialidase assay showed that most strains lacked sialidase activity despite some strains having a higher NS; thus, there is no association between sialidase and pathogenicity.

Resistance tests showed that *Gardnerella* was sensitive to antibiotics, including tetracycline, nitroimidazoles, macrolide and aminoglycosides. Meanwhile, the macrolide erythromycin resistance genes *ermX* and *lsaC*, and the tetracycline resistance genes *tetL* and *tetM* were also predicted in the ten strains. It is worth noting that seven out of the ten strains exhibited strong resistance (≥ 128 μg/mL) to metronidazole, which is the first line of treatment for BV in China. In addition to nitroreductase, YadH and McrA may also have roles in metronidazole resistance, with YadH involved in metronidazole excretion and McrA hydrolyzing the methyl-nitryl group from metronidazole (Lorca et al., 2007; Lubelski et al., 2007).

Additionally, we compared pathogenic genes of the prevalent strain in local China and the American type strain ATCC 14019. According to the phylogenetic analysis, strain ATCC 14019 belonged to the subtype C1, and possessed fairly thick biofilm and tobramycin resistance. Compared to strain ATCC 14019, the Chinese prevalent strains contained poor and incomplete TA systems with only antitoxin genes included, suggesting that the antitoxins may be essential to neutralize the toxins secreted by other microbes in the vaginal biome. The strains JNFY4, JNFY9, JNFY11, JNFY13, and JNFY14 without Abi-like proteins lack means of defense to a broad-range of bacteriocins produced by opponents, which probably is complemented by the single antitoxins (Kjos et al., 2010). Although there was no intact secretion system in *Gardnerella*, it was speculated that *Gardnerella* secreted toxic proteins to injure other bacteria, such as *Lactobacillus*, to enhance their survivability and displace the balance of the normal vaginal microflora. Strain ATCC 14019 encodes a methicillin resistance protein not found in the ten Chinese prevalent strains. Carbohydrate transport and metabolism genes of the 11 *Gardnerella* strains, including the genes associated with biofilm formation, were also analyzed and combined with biofilm thickness data (Figure 2 and Supplementary Figure 2). AraD, GalM, and GalT may be responsible for constructing biofilm, while FucP, MalA, and XylF are involved in monosaccharide transport. The additional monosaccharide units in the biofilm of strain JNFY14, arabinose and galactose hydrochloride, may play the role of connective elements which help to build a thicker biofilm.

**FIGURE 3:**
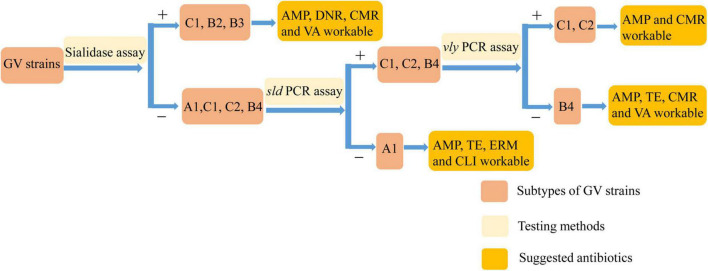
The diagnose pathway of *Gardnerella* strains.

Previous treatment standards involving clindamycin and metronidazole cannot account for all prevalent strains with sufficient efficacy to achieve complete recovery. Therefore, we need targeted treatments for diseases caused by different prevalent strains of *Gardnerella* to obtain the best results. Although the pathogenic genes of *Gardnerella* were previously characterized, the pathogenesis of BV is not yet understood. Through genomic and biochemical data analysis, we found that strains of subtype C could generally form biofilm, with varying degrees of antibiotic resistance (apart from invariable tobramycin resistance). Strain *G. swidsinskii* JNFY3 had a NS of 1, impaired ability to form biofilm, and lacked the *sld* and *lsaC* genes. *G. vaginalis* JNFY14 had the thickest biofilm, whereas strains JNFY17 and *G. piotii* JNFY15 rarely produced biofilm. On the whole, with the exception of strain JNFY14, the evolutionary trend appears to be the development of a progressively thicker biofilm from JNFY3 to ATCC 14019. All three strains (JNFY14, JNFY17, and JNFY15) showed some resistance to ciprofloxacin, gentamicin and tobramycin. Additionally, *G. piotii* JNFY15 lacked the *vly* gene and grew slower than other *Gardnerella* strains (Table 1). Based on this study, we constructed an initial database of *Gardnerella* prevalent strains in local China, which can be used as a reference to elucidate more accurate diagnostic pathways and treatments for BV (Table 1 and Figure 3).

## Data availability statement

The datasets presented in this study can be found in online repositories. The names of the repository/repositories and accession number(s) can be found in the article/Supplementary material.

## Ethics statement

The studies involving human participants were reviewed and approved by the Medical Ethics Committee of Jinan Maternity and Child Care Hospital. The patients/participants provided their written informed consent to participate in this study.

## Author contributions

LG, KZ, and RL conceptualized and designed the study. KZ and XZ analyzed the data. ML, KZ, KW, and HD accessed and verified the data. XJ collected samples from participants. KZ, FZ, and TL wrote the first draft of the manuscript. All authors contributed to drafting of the manuscript and read and approved the final version of the manuscript for submission, had full access to all data in the study, and had final responsibility for the decision to submit for publication.

## Footnotes

1
https://github.com/marbl/canu

2
https://github.com/hyattpd/Prodigal/wiki/introduction

3
http://www.pathogenomics.sfu.ca/islandviewer/upload/

4
http://www.ebi.ac.uk/interpro/search/sequence/

5
http://www.mgc.ac.cn/VFs/

6
http://antismash.secondarymetabolites.org/

7
http://tree.bio.ed.ac.uk/software/figtree/

## References

[B1] Amsel R. Totten P. A. Spiegel C. A. Chen K. C. S. Eschenbach D. Holmes K. K. (1983). Nonspecific vaginitis - diagnostic-criteria and microbial and epidemiologic associations. *Am. J. Med.* 74 14–22. 6600371 10.1016/0002-9343(83)91112-9

[B2] Anahtar M. N. Gootenberg D. B. Mitchell C. M. Kwon D. S. (2018). Cervicovaginal microbiota and reproductive health: The virtue of simplicity. *Cell Host Microbe* 23 159–168. 10.1016/j.chom.2018.01.013 29447695

[B3] Berger-Bachi B. Barberis-Maino L. Strassle A. Kayser F. H. (1989). FemA, a host-mediated factor essential for methicillin resistance in *Staphylococcus aureus*: Molecular cloning and characterization. *Mol. Gen. Genet.* 219 263–269. 10.1007/BF00261186 2559314

[B4] Bland C. Ramsey T. L. Sabree F. Lowe M. Brown K. Kyrpides N. C. (2007). CRISPR recognition tool (CRT): A tool for automatic detection of clustered regularly interspaced palindromic repeats. *BMC Bioinformatics* 8:209. 10.1186/1471-2105-8-209 17577412 PMC1924867

[B5] Cauci S. Culhane J. F. Di Santolo M. McCollum K. (2008). Among pregnant women with bacterial vaginosis, the hydrolytic enzymes sialidase and prolidase are positively associated with interleukin-1 beta. *Am. J. Obstet. Gynecol.* 198 132.e1–132.e7. 17714681 10.1016/j.ajog.2007.05.035

[B6] Cauci S. Thorsen P. Schendel D. E. Bremmelgaard A. Quadrifoglio F. Guaschino S. (2003). Determination of immunoglobulin A against *Gardnerella vaginalis* hemolysin, sialidase, and prolidase activities in vaginal fluid: Implications for adverse pregnancy outcomes. *J. Clin. Microbiol.* 41 435–438. 10.1128/JCM.41.1.435-438.2003 12517887 PMC149625

[B7] Chen Y. Han X. Guo P. Huang H. Wu Z. Liao K. (2018). Bacteramia caused by *Gardnerella vaginalis* in a cesarean section patient. *Clin. Lab.* 64 379–382. 29739103 10.7754/Clin.Lab.2017.171035

[B8] Clinical and Laboratory Standards Institute (2020a). *Methods for dilution antimicrobial susceptibility tests for bacteria that grow aerobically. Approved standard-eleventh edition, Document M07-A11.* Wayne, PA: CLSI.

[B9] Clinical and Laboratory Standards Institute (2020b). *Performance standards for antimicrobial susceptibility testing: Twenty-eighth informational supplement M100-S30.* Wayne, PA: CLSI.

[B10] Cohen C. R. Wierzbicki M. R. French A. L. Morris S. Newmann S. Reno H. (2020). Randomized trial of lactin-V to prevent recurrence of bacterial vaginosis. *N. Engl. J. Med.* 382 1906–1915. 32402161 10.1056/NEJMoa1915254 PMC7362958

[B11] Corfield T. (1992). Bacterial sialidases - roles in pathogenicity and nutrition. *Glycobiology* 2 509–521. 1472757 10.1093/glycob/2.6.509

[B12] Fettweis J. M. Serrano M. G. Brooks J. P. Edwards D. J. Girerd P. H. Parikh H. I. (2019). The vaginal microbiome and preterm birth. *Nat. Med.* 25 1012–1021. 31142849 10.1038/s41591-019-0450-2 PMC6750801

[B13] Francis V. I. Stevenson E. C. Porter S. L. (2017). Two-component systems required for virulence in *Pseudomonas aeruginosa*. *FEMS Microbiol. Lett.* 364:fnx104. 10.1093/femsle/fnx104 28510688 PMC5812489

[B14] Fredricks D. N. Fiedler T. L. Marrazzo J. M. (2005). Molecular identification of bacteria associated with bacterial vaginosis. *N. Engl. J. Med.* 353 1899–1911. 16267321 10.1056/NEJMoa043802

[B15] Gelber S. E. Aguilar J. L. Lewis K. L. Ratner A. J. (2008). Functional and phylogenetic characterization of Vaginolysin, the human-specific cytolysin from *Gardnerella vaginalis*. *J. Bacteriol.* 190 3896–3903. 10.1128/JB.01965-07 18390664 PMC2395025

[B16] Graham S. Howes C. Dunsmuir R. Sandoe J. (2009). Vertebral osteomyelitis and discitis due to *Gardnerella vaginalis*. *J. Med. Microbiol.* 58 1382–1384. 10.1099/jmm.0.007781-0 19541786

[B17] Greenwood J. R. Pickett M. J. (1980). Transfer of *Hemophilus vaginalis* gardner and dukes to a new genus, *Gardnerella*, *G. vaginalis* (gardner and dukes) comb nov. *Int. J. Syst. Bacteriol.* 30 170–178.

[B18] Gupta S. K. Padmanabhan B. R. Diene S. M. Lopez-Rojas R. Kempf M. Landraud L. (2014). ARG-ANNOT, a new bioinformatic tool to discover antibiotic resistance genes in bacterial genomes. *Antimicrob. Agents Chemother.* 58 212–220. 10.1128/AAC.01310-13 24145532 PMC3910750

[B19] Harwich M. D. Alves J. M. Buck G. A. Strauss J. F. Patterson J. L. Oki A. T. (2010). Drawing the line between commensal and pathogenic *Gardnerella vaginalis* through genome analysis and virulence studies. *BMC Genomics* 11:375. 10.1186/1471-2164-11-375 20540756 PMC2890570

[B20] He Q. Wang F. Liu S. H. Zhu D. Y. Cong H. J. Gao F. (2016). Structural and biochemical insight into the mechanism of Rv2837c from *Mycobacterium tuberculosis* as a c-di-NMP phosphodiesterase (vol 291, pg 3668, 2016). *J. Biol. Chem.* 291 14386–14387. 10.1074/jbc.M115.699801 27371563 PMC4933192

[B21] Hood R. D. Singh P. Hsu F. Guvener T. Carl M. A. Trinidad R. R. (2010). A type VI secretion system of *Pseudomonas aeruginosa* targets a toxin to bacteria. *Cell Host Microbe* 7 25–37. 10.1016/j.chom.2009.12.007 20114026 PMC2831478

[B22] Hyman R. W. Fukushima M. Diamond L. Kumm J. Giudice L. C. Davis R. W. (2005). Microbes on the human vaginal epithelium. *Proc. Natl. Acad. Sci. U.S.A.* 102 7952–7957. 15911771 10.1073/pnas.0503236102 PMC1142396

[B23] Janulaitiene M. Gegzna V. Baranauskiene L. Bulavaite A. Simanavicius M. Pleckaityte M. (2018). Phenotypic characterization of *Gardnerella vaginalis* subgroups suggests differences in their virulence potential. *PLoS One* 13:e0200625. 10.1371/journal.pone.0200625 30001418 PMC6042761

[B24] Janulaitiene M. Paliulyte V. Grinceviciene S. Zakareviciene J. Vladisauskiene A. Marcinkute A. (2017). Prevalence and distribution of *Gardnerella vaginalis* subgroups in women with and without bacterial vaginosis. *BMC Infect. Dis.* 17:394. 10.1186/s12879-017-2501-y 28583109 PMC5460423

[B25] Jarosik G. P. Land C. B. Duhon P. Chandler R. Jr. Mercer T. (1998). Acquisition of iron by *Gardnerella vaginalis*. *Infect. Immun.* 66 5041–5047. 9746616 10.1128/iai.66.10.5041-5047.1998 PMC108627

[B26] Jayaprakash T. P. Schellenberg J. J. Hill J. E. (2012). Resolution and characterization of distinct cpn60-based subgroups of *Gardnerella vaginalis* in the vaginal microbiota. *PLoS One* 7:e43009. 10.1371/journal.pone.0043009 22900080 PMC3416817

[B27] Kalvari I. Nawrocki E. P. Ontiveros-Palacios N. Argasinska J. Lamkiewicz K. Marz M. (2020). Rfam 14: Expanded coverage of metagenomic, viral and microRNA families. *Nucleic Acids Res.* 49 D192–D200. 10.1093/nar/gkaa1047 33211869 PMC7779021

[B28] Kim T. K. Thomas S. M. Ho M. F. Sharma S. Reich C. I. Frank J. A. (2009). Heterogeneity of vaginal microbial communities within individuals. *J. Clin. Microbiol.* 47 1181–1189. 19158255 10.1128/JCM.00854-08 PMC2668325

[B29] Kjos M. Snipen L. Salehian Z. Nes I. F. Diep D. B. (2010). The abi proteins and their involvement in bacteriocin self-immunity. *J. Bacteriol.* 192 2068–2076. 10.1128/JB.01553-09 20154137 PMC2849437

[B30] Laniewski P. Ilhan Z. E. Herbst-Kralovetz M. M. (2020). The microbiome and gynaecological cancer development, prevention and therapy. *Nat. Rev. Urol.* 17 232–250. 32071434 10.1038/s41585-020-0286-z PMC9977514

[B31] Lindahl G. Stalhammar-Carlemalm M. Areschoug T. (2005). Surface proteins of *Streptococcus agalactiae* and related proteins in other bacterial pathogens. *Clin. Microbiol. Rev.* 18 102–127. 15653821 10.1128/CMR.18.1.102-127.2005 PMC544178

[B32] Lorca G. L. Barabote R. D. Zlotopolski V. Tran C. Winnen B. Hvorup R. N. (2007). Transport capabilities of eleven gram-positive bacteria: Comparative genomic analyses. *Biochim. Biophys. Acta* 1768 1342–1366. 17490609 10.1016/j.bbamem.2007.02.007 PMC2592090

[B33] Lubelski J. Konings W. N. Driessen A. J. (2007). Distribution and physiology of ABC-type transporters contributing to multidrug resistance in bacteria. *Microbiol. Mol. Biol. Rev.* 71 463–476. 10.1128/MMBR.00001-07 17804667 PMC2168643

[B34] McGregor J. A. French J. I. (2000). Bacterial vaginosis in pregnancy. *Obstet. Gynecol. Surv.* 55(5 Suppl. 1), S1–S19. 10804540 10.1097/00006254-200005001-00001

[B35] Menard J. P. Mazouni C. Salem-Cherif I. Fenollar F. Raoult D. Boubli L. (2010). High vaginal concentrations of *Atopobium vaginae* and *Gardnerella vaginalis* in women undergoing preterm labor. *Obstet. Gynecol.* 115 134–140. 10.1097/AOG.0b013e3181c391d7 20027045

[B36] Neri P. Salvolini S. Giovannini A. Mariotti C. (2009). Retinal vasculitis associated with asymptomatic *Gardnerella vaginalis* infection: A new clinical entity. *Ocul. Immunol. Inflamm.* 17 36–40. 10.1080/09273940802491876 19294572

[B37] Nugent R. P. Krohn M. A. Hillier S. L. (1991). Reliability of diagnosing bacterial vaginosis is improved by a standardized method of gram stain interpretation. *J. Clin. Microbiol.* 29 297–301. 10.1128/jcm.29.2.297-301.1991 1706728 PMC269757

[B38] Patterson J. L. Stull-Lane A. Girerd P. H. Jefferson K. K. (2010). Analysis of adherence, biofilm formation and cytotoxicity suggests a greater virulence potential of *Gardnerella vaginalis* relative to other bacterial-vaginosis-associated anaerobes. *Microbiology* 156 392–399. 10.1099/mic.0.034280-0 19910411 PMC2890091

[B39] Peebles K. Velloza J. Balkus J. E. McClelland R. S. Barnabas R. V. (2019). High global burden and costs of bacterial vaginosis: A systematic review and meta-analysis. *Sex. Transm. Dis.* 46 304–311. 30624309 10.1097/OLQ.0000000000000972

[B40] Pleckaityte M. Janulaitiene M. Lasickiene R. Zvirbliene A. (2012). Genetic and biochemical diversity of *Gardnerella vaginalis* strains isolated from women with bacterial vaginosis. *FEMS Immunol. Med. Microbiol.* 65 69–77. 10.1111/j.1574-695X.2012.00940.x 22309200

[B41] Punsalang A. P. Jr. Sawyer W. D. (1973). Role of pili in the virulence of *Neisseria gonorrhoeae*. *Infect. Immun.* 8 255–263. 4200266 10.1128/iai.8.2.255-263.1973 PMC422841

[B42] Rainey F. A. Ward-Rainey N. Kroppenstedt R. M. Stackebrandt E. (1996). The genus *Nocardiopsis* represents a phylogenetically coherent taxon and a distinct actinomycete lineage: Proposal of *Nocardiopsaceae* fam. nov. *Int. J. Syst. Bacteriol.* 46 1088–1092. 10.1099/00207713-46-4-1088 8863440

[B43] Rosca A. S. Castro J. Sousa L. G. V. Cerca N. (2020). *Gardnerella* and vaginal health: The truth is out there. *FEMS Microbiol. Rev.* 44 73–105. 10.1093/femsre/fuz027 31697363

[B44] Rottini G. Dobrina A. Forgiarini O. Nardon E. Amirante G. A. Patriarca P. (1990). Identification and partial characterization of a cytolytic toxin produced by *Gardnerella vaginalis*. *Infect. Immun.* 58 3751–3758. 10.1128/iai.58.11.3751-3758.1990 2228246 PMC313724

[B45] Sivadon-Tardy V. Roux A. L. Piriou P. Herrmann J. L. Gaillard J. L. Rottman M. (2009). *Gardnerella vaginalis* acute hip arthritis in a renal transplant recipient. *J. Clin. Microbiol.* 47 264–265. 10.1128/JCM.01854-08 19020054 PMC2620833

[B46] Swidsinski A. Mendling W. Loening-Baucke V. Ladhoff A. Swidsinski S. Hale L. P. (2005). Adherent biofilms in bacterial vaginosis. *Obstet. Gynecol.* 106(5 Pt 1), 1013–1023. 16260520 10.1097/01.AOG.0000183594.45524.d2

[B47] Vaneechoutte M. Guschin A. Van Simaey L. Gansemans Y. Van Nieuwerburgh F. Cools P. (2019). Emended description of *Gardnerella vaginalis* and description of *Gardnerella leopoldii* sp. nov., *Gardnerella piotii* sp. nov. and *Gardnerella swidsinskii* sp. nov., with delineation of 13 genomic species within the genus *Gardnerella*. *Int. J. Syst. Evol. Microbiol.* 69 679–687. 10.1099/ijsem.0.003200 30648938

[B48] Walker B. J. Abeel T. Shea T. Priest M. Abouelliel A. Sakthikumar S. (2014). Pilon: An integrated tool for comprehensive microbial variant detection and genome assembly improvement. *PLoS One* 9:e112963. 10.1371/journal.pone.0112963 25409509 PMC4237348

[B49] Workowski K. A. Berman S. Centers for Disease Control and Prevention. (2010). Sexually transmitted diseases treatment guidelines, 2010. *MMWR Recomm. Rep.* 59 1–110. 16888612

[B50] Yeoman C. J. Yildirim S. Thomas S. M. Durkin A. S. Torralba M. Sutton G. (2010). Comparative genomics of *Gardnerella vaginalis* strains reveals substantial differences in metabolic and virulence potential. *PLoS One* 5:e12411. 10.1371/journal.pone.0012411 20865041 PMC2928729

[B51] Zhang Y. Rochefort D. (2013). Fast and effective paper based sensor for self-diagnosis of bacterial vaginosis. *Anal. Chim. Acta* 800 87–94. 10.1016/j.aca.2013.09.032 24120172

