# Supplementary material for: Antibiotic resistance and pathogenicity assessment of various Gardnerella sp. strains in local China
Source: Front Microbiol. 2022 Sep 26;13:1009798. doi: 10.3389/fmicb.2022.1009798 (PMC9549249; doi:10.3389/fmicb.2022.1009798)
Supplement: Supplementary file 1 [file Data_Sheet_1.pdf]

Supplementary information

Antibiotic resistance and pathogenicity assessment of various *Gardnerella* sp. strains in local China

Running title: **Antibiotic resistance and pathogenicity of *Gardnerella***

Kundi Zhang[a], Mengyao Lu[a], Xiaoxuan Zhu[a], Kun Wang[b], Xuemei Jie[b], Tan Li[c], , Hongjie Dong[a], Rongguo Li[b], Fengyu Zhang[a,*], Lichuan Gu[a,*]

State Key Laboratory of Microbial Technology, Shandong University, Qingdao 266237, Shandong, China [a]; Jinan Key Laboratory of Female Reproductive Tract Infection, Jinan Genital Tract Microecological Clinical Laboratory, Jinan Maternity and Child Care Hospital Affiliated to Shandong First Medical University, Jinan 250001, China[b]; Faculty of Health Sciences, Cumming School of Medicine, 3330 Hospital Dr NW, Calgary, AB T2N 4N1, Canada[c]

*Correspondence to:

Dr. Fengyu Zhang, State Key Laboratory of Microbial Technology, Shandong University, Qingdao 266237, Shandong, China, zfengyu@mail.sdu.edu.cn.

Prof Lichuan Gu, State Key Laboratory of Microbial Technology, Shandong University, Qingdao 266237, Shandong, China, lcgu@sdu.edu.cn.

I. Supplemental Figures

II. Supplemental Tables

I. Supplemental Figures

Figure S1. Phylogenomic tree of *Gardnerella* strains. A proteomic tree was constructed based on the genome sequences of 21 *Gardnerella* strains. The tree was built by the neighbor-joining method, and bootstrap value above 90 are shown at node points, based on 1,000 replicates.

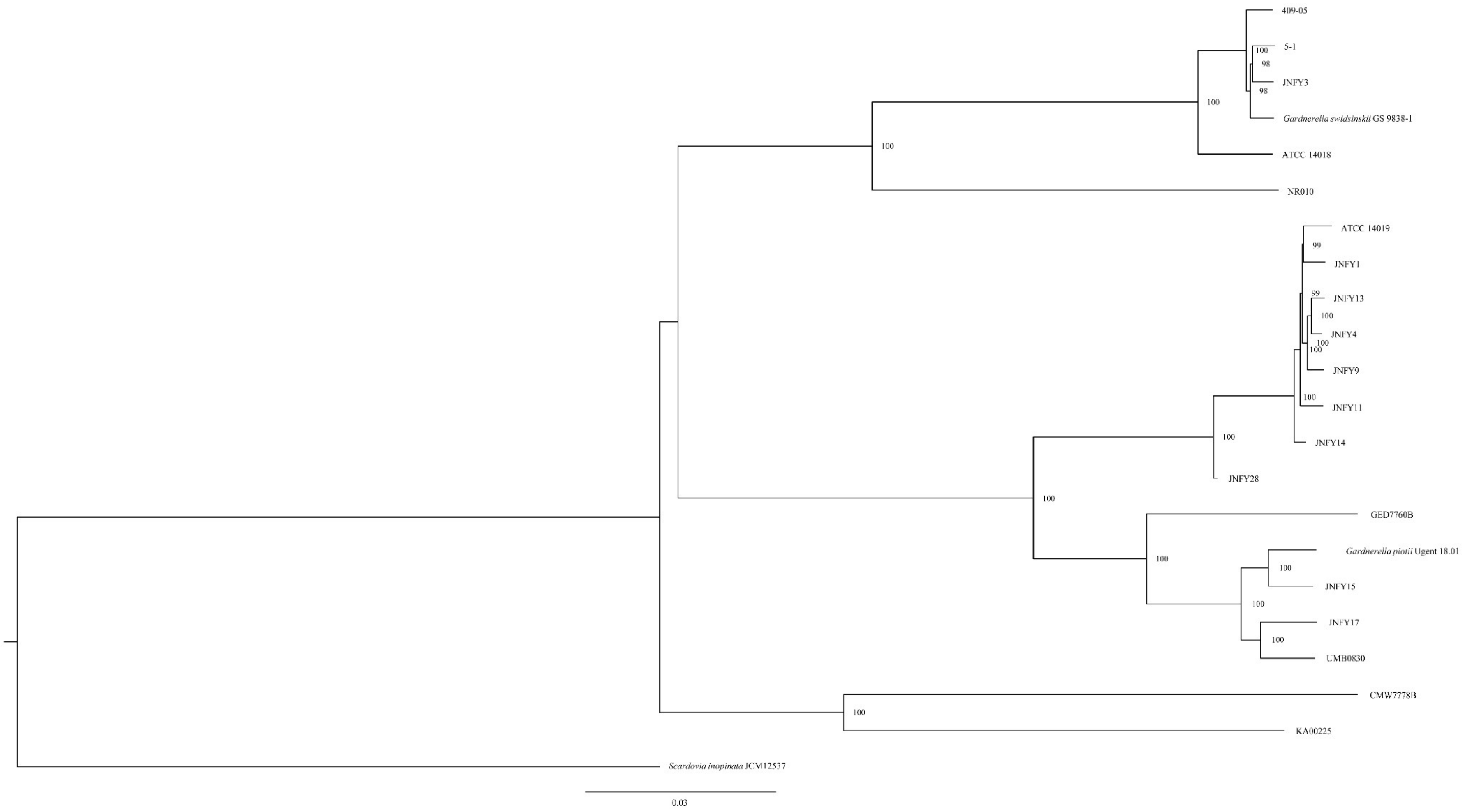
409-05
5-1
JNFY3
Gardnerella swidsinskii GS 9838-1
ATCC 14018
NR010
ATCC 14019
JNFY1
JNFY13
JNFY4
JNFY9
JNFY11
JNFY14
JNFY28
GED7760B
Gardnerella piotii Ugent 18.01
JNFY15
JNFY17
UMB0830
CMW7778B
KA00225
Scardovia inopinata JCM12537
0.03

Figure S2. Crystal violet staining of the biofilm formed by *Gardnerella* strains.

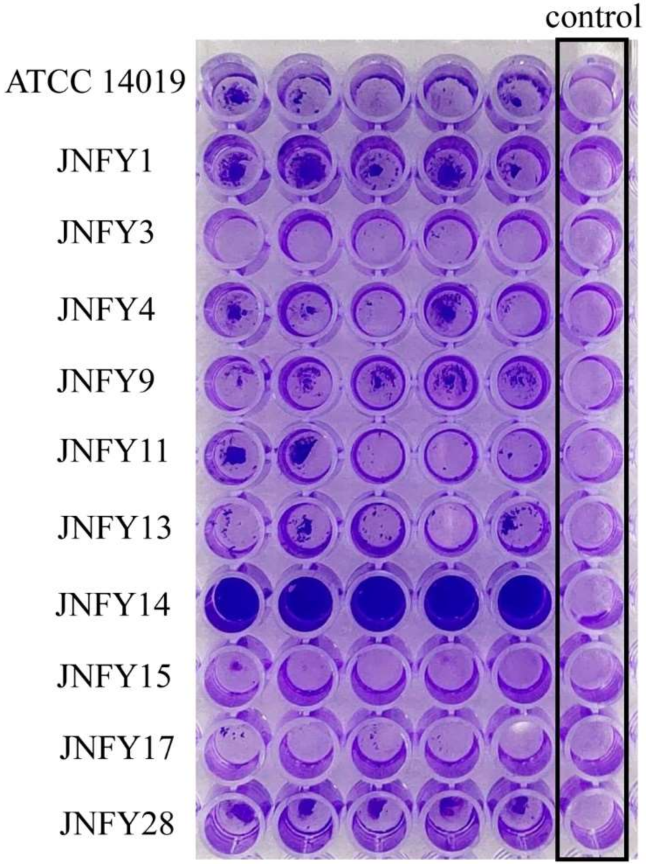

II. Supplemental Tables

Table S1. PCR primers used in this study.

Primers	Sequence(5’-3’)
16S27F	GAGTTTGATCCTGGCTCAG
16S1492R	AGAAAGGAGGTGATCCAGCC
cpn60F	ATGGCAAAGATTATTGCCTATGAAG
cpn60R	GCATTCTGTAGAGCAGAACGAGT
vlyF	GTACGATTCTGCAAGCGCACAAAGC
vlyR	CCTTCCCAAGCGCGAGAACGC
sldF	ATGGAACGTCGTTCAACGAAG
sldR	GATACGCGTTTTATGTCTCTTGC

Table S2. Carbohydrate transport and metabolism genes comparison of the *Gardnerella* strains.

Gene	Annotation	ATCC 14019	JNFY1	JNFY3	JNFY4	JNFY9	JNFY11	JNFY13	JNFY14	JNFY15	JNFY17	JNFY28
AfuC	Alpha-L-fucosidase	1	1	0	1	1	1	1	1	0	0	0
AraD	Ribulose-5-phosphate 4-epimerase/Fuculose-1-phosphate aldolase	0	1	0	1	1	1	1	1	0	0	0
FucP	Fucose permease	0	1	0	1	1	1	1	1	0	0	0
GalM	Galactose mutarotase or related enzyme	0	1	0	1	1	1	1	1	0	0	0
GalT	Galactose-1-phosphate uridylyltransferase	2	1	0	1	1	1	1	1	0	0	0
LacZ	Beta-galactosidase/beta-glucuronidase	2	2	0	2	2	2	2	2	0	0	0
MglA	ABC-type sugar transport system, ATPase component	0	4	1	4	4	4	4	4	0	0	0
XylB	Sugar (pentulose or hexulose) kinase	0	2	0	2	2	2	2	2	0	0	0
XylF	ABC-type xylose transport system, periplasmic component	0	1	0	2	2	1	2	2	0	0	0
NagC	Sugar kinase of the NBD/HSP70 family, may contain an N-terminal HTH domain	0	7	1	8	7	8	8	7	4	2	2
UgpA	ABC-type sugar transport system, permease component	8	8	3	9	8	10	9	8	3	4	4
UgpB	ABC-type glycerol-3-phosphate	3	8	4	9	8	10	9	8	3	4	4

	transport system, periplasmic component											
UgpE	ABC-type glycerol-3-phosphate transport system, permease component	3	7	2	8	7	9	8	7	2	3	3

Table S3. Common virulence factors of the ten *Gardnerella* epidemic strains.

Virulence factor	Related gene	Product
Adhesion		
ABC transporter ATP-binding protein AatC	*aatC*	ABC transporter
Polyphosphate kinase(type IV pili)	*ppK*	polyphosphate kinase 1
Surface-anchored pilus proteins	*sapD*	actinobacterial surface-anchored protein domain
Elongation factor Tu	*tuf*	small GTP-binding protein domain
Trigger factor	*tig/ropA*	trigger factor
Immunogenic lipoprotein A(IlpA)	*IlpA*	NLPA lipoprotein
Listeria adhesion protein	*lap*	Aldehyde dehydrogenase family
Iron uptake		
Iron related(ABC transporter)	*fbpC*	ABC transporter
Putative iron transport system ATP-binding protein	*ciuD*	phosphate ABC transporter, ATP-binding protein
ABC transporter ATP-binding protein	*irtB*	FeS assembly ATPase SufC
Ferrous iron transport	*sitC*	anchored repeat-type ABC transporter, permease subunit
Transcriptional repressor of iron-responsive genes	*fur*	Ferric uptake regulator family
Iron(III)	*fbpC,hitC*	ABC transporter
Secretion system		
preprotein translocase subunit SecA (Accessory secretion factor)	*secA2*	preprotein translocase, SecA subunit
type III secretion system ATPase(T3SS)	*DVUA0119*	ATP synthase F1, alpha subunit
type III secretion system ATPase(T3SS)	*ssaN*	transcription termination factor Rho
Dot/Icm type IV secretion system effector	*lirB*	Cyclophilin type peptidyl-prolyl cis-trans

LirB(Dot/Icm)		isomerase
T6SS-II(CVF861)	protein disaggregation chaperone	ATP-dependent chaperone protein ClpB
Invasion		
Lipoprotein promoting entry protein	*lpeA*	actinobacterial surface-anchored protein domain
P60 extracellular protein, invasion associated protein Iap	*iap/cwhA*	NlpC/P60 family
Stress protein		
endopeptidase Clp ATP-binding chain C(ClpC)	*clpC*	ATPase family associated with various cellular
ATP-dependent Clp protease proteolytic subunit(ClpP)	*clpP*	Clp protease
Antibiotic resistance		
daunorubicin resistance ABC transporter ATP-binding subunit	*ddrA*	ABC transporter
Regulation of virulence-associated genes		
ppGpp synthetase, RelA/SpoT family	*relA*	RelA/SpoT family protein
S-ribosylhomocysteinase(Autoinducer-2)	*luxS*	S-Ribosylhomocysteinase (LuxS)
two-component system response regulator(DevR/S)	*devR/dosR*	Response regulator receiver domain
membrane-bound transcriptional regulator LytR (Polysaccharide capsule)	*lytR*	cell envelope-related function transcriptional attenuator common domain

Table S4. Partially-shared virulence factors of the ten *Gardnerella* epidemic strains.

Virulence factor	Related gene	Product	Strains lacking the gene
Adhesion			
tight adherence protein A (Flp pili)	*tadA*	Type II/IV secretion system protein	JNFY3, JNFY13, JNFY28
SpaD-type pili	*srtB*	sortase	JNFY28
Type IV pili response regulator PilH	*pilH*	Response regulator receiver domain	JNFY15, JNFY17
Iron uptake			
phospho-2-dehydro-3-deoxyheptonate aldolase (vulnibactin)	*VVA1298*	3-deoxy-7-phosphoheptulonate synthase	JNFY3
Haemophilus iron transport locus	*hitC,hitC*	ABC transporter	JNFY3
putative iron ABC transport system,	*hmuV*	ABC transporter	JNFY3
Yersiniabactin (Ybt)	*ybtQ*	ABC transporter	JNFY3、JNFY9、JNFY15、JNFY17
Acinetobactin	*bauE,barA*	ABC transporter	JNFY3
Magnesium uptake			
Magnesium transport ATPase, P-type 2	*mgtB*	HAD ATPase, P-type, family IC	JNFY3
Secretion system			
ESX-1	*eccA1*	Holliday junction DNA helicase RuvB	JNFY3、JNFY15、JNFY17
Coxiella Dot/Icm type IVB secretion system translocated effector (Dot/Icm)	*coxH2,coxFIC1*	ribosomal-protein-alanine acetyltransferase	JNFY3、JNFY15
Type VII secretion system	*essC*	FtsK/SpoIIIE family	JNFY15

T6SS-III (CVF862)	periplasmic sugar-binding protein	Periplasmic binding protein domain	JNFY15 、 JNFY17 、 JNFY28
Toxin			
vaginolysin	*HMPREF0424_0103*	Thiol-activated cytolysin	JNFY15
hemolysin A (Hemolysin)	*CLD_2757*	TlyA family rRNA methyltransferase	JNFY15、JNFY17
HlyC/CorC family (Hemolysin)	*CLK_2301*	CBS domain	JNFY3
hemolysin B (Alpha-hemolysin)	*hlyB*	ABC transporter	JNFY15、JNFY17
exo-alpha-sialidase (sialidase)	*nanJ*, *nanI*	BNR repeat-like domain	JNFY3
pyrroline-5-carboxylate reductase (Proline synthesis)	*proC*	pyrroline-5-carboxylate reductase	JNFY15
Endotoxin			
Fused lipid transporter subunits of ABC superfamily (LOS)	*msbA*	ABC transporter	JNFY3
Putative formyl transferase (LPS)	*wbmU*	methionyl-tRNA formyltransferase	JNFY15、JNFY17
Immune vasion			
lipooligosaccharide biosynthesis galactosyltransferase, putative (LOS)	*CJE1280*	Glycosyl transferase family 2	JNFY3、JNFY15、JNFY17
phosphoglucosamine mutase (Exopolysaccharide)	*mrsA/glmM*	phosphoglucosamine mutase	JNFY3
exopolysaccharide biosynthesis protein, glycosyl transferase	*eps15*	Glycosyl transferases group 1	JNFY3、JNFY15、JNFY17

(Capsule)			
RgpG (Capsule)	*SGO_1723*	phospho-N-acetylmuramoyl-pentapeptide-transferase	JNFY3、JNFY15、JNFY17
dTDP-4-dehydrorhamnose 3,5-epimerase or related enzyme (Capsule)	*STER_1223*	dTDP-4-dehydrorhamnose 3,5-epimerase	JNFY3
Regulation			
lysozyme	*lytC*	Glycosyl hydrolases family 25	JNFY15 、 JNFY17 、 JNFY28
WhiB family transcriptional regulator (WhiB3)	*whiB3*	Transcription factor WhiB	JNFY3
RNA polymerase sigma factor SigA (Sigma A)	*sigA/rpoV*	RNA polymerase sigma factor RpoD	JNFY15、JNFY17
DNA-binding response regulator PhoP (PhoP/R)	*phoP*	Response regulator receiver domain	JNFY3
two-component system response phosphate sensor kinase, PhoR (PhoP/R)	*phoR*	His Kinase A (phospho-acceptor) domain	JNFY3 、 JNFY13 、 JNFY15、JNFY17
two component transcriptional regulator (RegX3)	*regX3*	Response regulator receiver domain	JNFY3
GTP pyrophosphokinase (RelA)	*relA*	Region found in RelA / SpoT proteins	JNFY3

Table S5. Unique virulence factors of strains JNFY3 and JNFY4.

Strain	Virulence factor	Related gene
JNFY3	allantoate amidohydrolase (Allantion utilization)	*allC*
	Allantoinase (Allantion utilization)	*allB*
	Glycosyltransferase (Capsule I)	*BCAL3234*
	putative polysaccharide permease protein (Capsule)	*SGO_1023*
	choline permease (LOS)	*licB*
	aggregation substance (AS)	*EF0149*
JNFY4	Bartonella effector protein BepA (VirB/VirD4 type IV secretion system)	*bepA*
